# The shared genetic architecture of smoking behaviours and psychiatric disorders: evidence from a population-based longitudinal study in England

**DOI:** 10.1186/s12863-023-01131-8

**Published:** 2023-05-30

**Authors:** Olesya Ajnakina, Andrew Steptoe

**Affiliations:** 1 Department of Behavioural Science and Health, Institute of Epidemiology and Health Care, University College London, 16 De Crespigny Park, London, SE5 8AF UK; 2 Department of Biostatistics & Health Informatics, Institute of Psychiatry, Psychology and Neuroscience, King’s College London, University of London, 1-19 Torrington Place, London, WC1E 7HB UK

**Keywords:** Polygenic scores, Schizophrenia, Bipolar disorders, Major depressive disorders, Intelligence, Genome-wide association studies, Smoking initiation, Smoking cessation, Nicotine dependence, Older adults

## Abstract

**Background:**

Considering the co-morbidity of major psychiatric disorders and intelligence with smoking, to increase our understanding of why some people take up smoking or continue to smoke, while others stop smoking without progressing to nicotine dependence, we investigated the genetic propensities to psychiatric disorders and intelligence as determinants of smoking initiation, heaviness of smoking and smoking cessation in older adults from the general population.

**Results:**

Having utilised data from the English Longitudinal Study of Ageing (ELSA), our results showed that one standard deviation increase in MDD-PGS was associated with increased odds of being a moderate-heavy smoker (odds ratio [OR] = 1.11, SE = 0.04, 95%CI = 1.00-1.24, *p* = 0.028). There were no other significant associations between SZ-PGS, BD-PGS, or IQ-PGS and smoking initiation, heaviness of smoking and smoking cessation in older adults from the general population in the UK.

**Conclusions:**

Smoking is a behaviour that does not appear to share common genetic ground with schizophrenia, bipolar disorders, and intelligence in older adults, which may suggest that it is more likely to be modifiable by smoking cessation interventions. Once started to smoke, older adults with a higher polygenic predisposition to major depressive disorders are more likely to be moderate to heavy smokers, implying that these adults may require targeted smoking cessation services.

**Supplementary Information:**

The online version contains supplementary material available at 10.1186/s12863-023-01131-8.

## Background

Cigarette smoking is the leading preventable cause of disability and mortality [1, 2], particularly among older adults [3]. Indeed, it has been shown that 70% of smoking-induced excess mortality occurs in people over the age of 60 [4]. Though smoking prevalence is highest in young and middle-aged adults in most countries, it remains highly prevalent in older ages. For example, around 16% of people who are aged 65 or over are reported to be smokers [5], despite the well-documented risks associated with it. These include a substantially higher risk of heart failure, symptomatic peripheral vascular disease, myocardial infarction, transient ischaemic attack or stroke, and chronic renal failure when compared with their ex-smoker counterparts, even when compared with those who quit smoking in old age [6, 7]. The risk for these health conditions is three-fold greater in adults who smoke between 1 and 4 cigarettes a day when compared to adults who never smoked [6]. Therefore, there is an emergent need to ascertain why some people take up smoking or continue to smoke, while others stop smoking without progressing to nicotine dependence.

Twin and family studies have consistently demonstrated that smoking-related behaviours have a significant genetic component, with an estimated heritability of 0.50–0.70 for smoking initiation and 0.60 for nicotine dependence [8]. More recently, polygenic scores (PGSs), which reflect a mathematical aggregate of risk conferred by many genetic variants of small effect [9], have revealed that smoking initiation, heaviness of smoking and smoking cessation are influenced by separate sets of common genetic markers [10]. This suggests that genetic differences within individuals have an important role in these smoking-related behaviours [11].

Cigarette smoking is a frequent co-morbidity of major psychiatric disorders, such as schizophrenia, bipolar disorders, and major depression [12]. For example, it is well known that a large proportion of schizophrenia patients tend to be heavy smokers [13]. Nonetheless, the underlying nature of this comorbidity is not well understood [14]. Because high rates of smoking are also observed among first-degree relatives of patients with a diagnosis of schizophrenia and bipolar disorders [15, 16], it has been postulated that genetic factors linked to these disorders may contribute to smoking-related behaviours thereby increasing the risk of addiction [17–20]. Indeed, recent genome-wide association studies showed that smoking initiation and nicotine dependence have a shared genetic component with schizophrenia and bipolar disorders [18–20], which in turn are genetically correlated with major depression and intelligence [21–23]. Previous studies further suggested that a higher polygenic predisposition to intelligence may contribute to a better understanding of the health consequences of cigarette smoking thereby reducing the likelihood initiate smoking [24–26]. Cumulatively, it may be argued that smoking-related behaviours in the general population might arise from genetic factors associated with major psychiatric conditions and intelligence. However, because most research on cigarette smoking is focused on either adolescence or young adults [27], there is a substantial gap in our knowledge of the role the common genetic markers play in smoking-related behaviours in older adults.

Using PGSs can aid in elucidating whether co-morbidity relationships between cigarette smoking and severe mental illnesses, such as schizophrenia, bipolar disorders, and major depression reflect shared genetics [20]. By applying the PGSs methodology to individuals who do not have psychiatric disorders, this approach can provide a separation of the effect of the diseases themselves from those of the underlying genetic risk factors. Therefore, using the PGSs approach, the present study aimed to investigate if schizophrenia, bipolar disorders, major depressive disorders, and intelligence have shared genetic liability with smoking initiation, heaviness of smoking and smoking cessation in older adults from the general population. We hypothesised that PGSs for these major psychotic disorders will be significantly associated with smoking-related behaviours in the general population of older adults.

## Results

### Study participants

The baseline sample characteristics of ELSA participants are presented in Table 1. The sample comprised 6185 individuals with a mean age of 64.8 years old (standard deviation (SD) = 9.3, range = 50–101, median = 63, IQR = 57–71); 47.8% (*n* = 2959) were men. Of the entire sample, 62.3% (*n* = 3874) initiated smoking at some point in their lives; of these, 86.5% (*n* = 3436) were light smokers, and 13.5% (*n* = 538) were moderate to heavy smokers. Of all those participants who reported having smoked, 22.5% (*n* = 892) did not cease smoking at the time of the interview. Compared to non-smokers, of those older adults who reported to be smokers (former or current), a higher portion were men, self-reported to have poor health and had low accumulated wealth.

**Table 1:** Participant characteristics by smoking behaviours

Participant characteristics	Smoking initiation			Moderate-heavy smokers			Smoking cessation	
	Never	Smokers		No	Yes		Stopped smoking	Still smoke
	n = 2408 (37.7%)	n = 3974 (62.3%)		n = 3436 (86.5%)	n = 538 (13.5%)		n = 3082 (77.5%)	n = 892 (22.5%)
Age (years)	64.6 (9.4)	65.5 (9.5)		66.2 (9.5)	60.8 (7.6)		66.4 (9.6)	62.4 (8.2)
Gender, men	905 (37.6)	2142 (53.9)		1885 (54.9)	257 (47.8)		1716 (55.7)	426 (47.8)
Poor self-rated health	398 (16.5)	1006 (25.3)		821 (23.9)	185 (34.4)		694 (22.5)	312 (35.0)
Currently married	1707 (70.9)	2745 (69.1)		2415 (70.3)	330 (61.3)		2205 (71.5)	540 (60.5)
Accumulated wealth								
High	982 (40.8)	1333 (33.5)		1239 (36.1)	94 (17.5)		1164 (37.8)	169 (18.9)
Mid	784 (32.6)	1175 (29.6)		1033 (30.1)	142 (26.4)		943 (30.6)	232 (26.0)
Low	642 (26.7)	1466 (36.9)		1164 (33.9)	302 (56.1)		975 (31.6)	491 (55.0)
Completed schooling (years)	14.3 (3.7)	13.7 (3.7)		13.8 (3.8)	13.2 (3.5)		13.9 (3.8)	13.0 (3.5)

The values in parenthesis are standard deviations for continuous variables and percentages for categorical variables

### Smoking initiation

Results from associations between PGSs and the likelihood to initiate cigarette smoking in men and women from the general population in the UK are provided in Table 2. One standard deviation increase in SZ-PGS was not significantly associated with a likelihood of smoking initiation (odds ratio [OR] = 1.01, SE = 0.03, 95%CI = 0.95–1.06, *p* = 0.817). Similarly, there were no statistically significant associations between initiating cigarette smoking and BD-PGS, MDD-PGS or IQ-PGS in older adults.

**Table 2:** Associations between polygenic scores and likelihood to initiate cigarette smoking among older adults from the general population in the UK

Polygenic scores			Smoking initiation		
		OR	SE	95%CI	*p*
**SZ-PGS**		1.01	0.03	0.95–1.06	0.817
**BD-PGS**		1.00	0.03	0.94–1.05	0.888
**MDD-PGS**		0.99	0.03	0.94–1.05	0.835
**IQ-PGS**		1.00	0.03	0.95–1.06	0.895

SZ-PGS, polygenic score for schizophrenia; BD-PGS, polygenic score for bipolar disorder; MDD-PGS, polygenic score for major depressive disorder; IQ-PGS, polygenic score for intelligence; OR, odds ratio’ SE, standard error; CI, confidence intervals

### The heaviness of cigarettes smoking

**Fig. 1:**
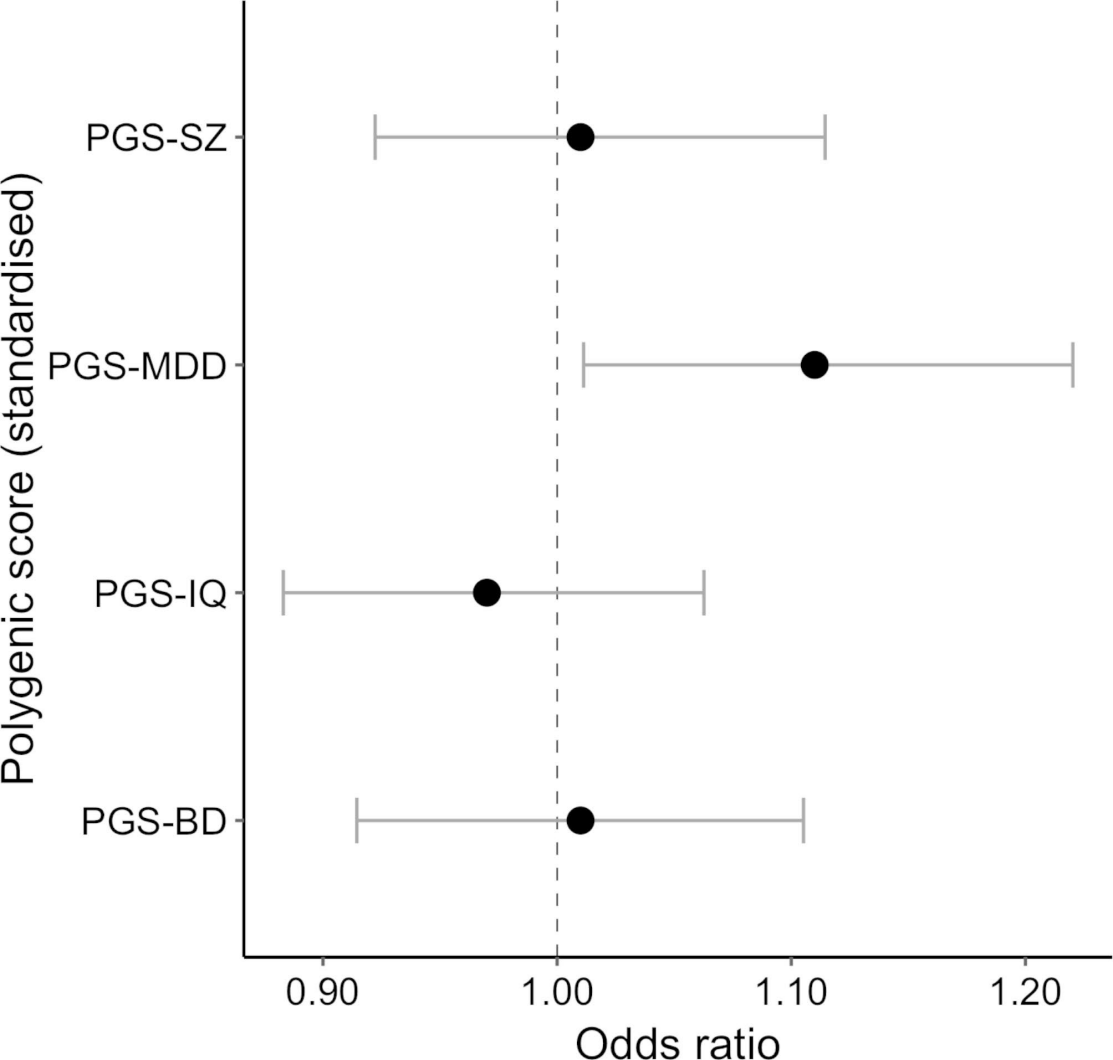
presents the results from associations between polygenic scores and the likelihood to smoke cigarettes heavily among men and women from the general population in the UK.

Associations between polygenic scores and the likelihood of heavy cigarette smoking among men and women from the general population in the UK are presented in Table 3. One standard deviation increase in MDD-PGS was associated with increased odds of being a moderate-heavy smoker (OR = 1.11, SE = 0.05, 95%CI = 1.00-1.24, *p* = 0.028). These results are further depicted in Fig. 1. There were no significant relationships of the heaviness of cigarette smoking with SZ-PGS, BD-PGS and IQ-PGS in our sample.

**Table 3:** Associations between polygenic scores and heaviness of cigarette smoking among older adults from the general population in the UK

Polygenic scores			Heaviness of cigarettes smoking			
			OR	SE	95%CI	*p*
**SZ-PGS**			1.01	0.05	0.91–1.12	0.776
**BD-PGS**			1.00	0.05	0.90–1.12	0.913
**MDD-PGS**			1.11	0.05	1.00-1.24	0.028
**IQ-PGS**			0.97	0.05	0.88–1.07	0.501

SZ-PGS, polygenic score for schizophrenia; BD-PGS, polygenic score for bipolar disorder; MDD-PGS, polygenic score for major depressive disorder; IQ-PGS, polygenic score for intelligence; OR, odds ratio; SE, standard error; CI, confidence intervals

### Smoking cessation

Associations between polygenic scores and the likelihood to stop cigarette smoking among men and women from the general population in the UK are presented in Table 4. One standard deviation increase in SZ-PGS was not significantly associated with a likelihood to stop cigarette smoking (OR = 1.01, SE = 0.04, 95%CI = 0.91–1.08, *p* = 0.990). Similarly, there were no statistically significant associations between smoking cessation and BD-PGS, MDD-PGS, or IQ-PGS in older adults.

**Table 4:** Associations between polygenic scores and likelihood to stop cigarette smoking among older adults from the general population in the UK

Polygenic scores		Smoking cessation			
		OR	SE	95%CI	*p*
**SZ-PGS**		1.00	0.04	0.92–1.08	0.990
**BD-PGS**		0.99	0.04	0.91–1.08	0.817
**MDD-PGS**		1.06	0.04	0.97–1.15	0.168
**IQ-PGS**		1.00	0.04	0.93–1.09	0.896

SZ-PGS, polygenic score for schizophrenia; BD-PGS, polygenic score for bipolar disorder; MDD-PGS, polygenic score for major depressive disorder; IQ-PGS, polygenic score for intelligence; OR, odds ratio; SE, standard error; CI, confidence intervals

## Discussion

To our knowledge, this is the first study to have investigated relationships of smoking initiation, heaviness of smoking and smoking cessation with aggregate measures of genetic risk for major psychiatric disorders and intelligence in older adults from the general population in the UK. Cumulatively, our results demonstrate that smoking is a behaviour that does not appear to share common genetic markers with schizophrenia, bipolar disorders, and intelligence. Whereas moderate to heavy smoking is associated with the additive effects of genetic factors associated with major depressive disorders, reiterating an assertion that the genetic risks captured with polygenic scores influence response to nicotine and not the propensity to initiate smoking [12].

Two-thirds of all adults in our cohort reported smoking at some point during their lives, with 13.5% of these being moderate to heavy smokers. Stopping smoking in old age have several proven benefits including a reduced morbidity and lower mortality risk. For example, it has been shown that having stopped smoking at the age of 65 and over led to a statistically significant reduction in risk of all-cause mortality in the following 1–2 years and approached that of never-smokers after 15–20 years [28, 29]. This recognition ignited significant efforts in encouraging the public in the UK to stop smoking by offering free smoking cessation services staffed by expert advisers as well as targeting workplace tobacco control interventions [30]. Although surveys have indicated that a large proportion of elderly smokers would like to quit [3], our results showed that 22.5% of adults with an average age of 64.8 years old remained smokers, which is consistent with the national estimates of smoking-related behaviours [1].

It was previously shown that smoking initiation and schizophrenia diagnosis shared a substantial component of common genetic variation shared between [19, 20]. However, in the present study, older adults who were smokers did not differ in their polygenic predisposition to schizophrenia from adults who never smoked. We further considered that high genetic predisposition either to bipolar disorders, major depressive disorders, or intelligence might be associated with smoking initiation in older adults. Once again, our findings were negative. In contrast to our findings, using a sample of *n* = 144,609 Icelandic individuals, it was previously shown that there was a common genetic variation that was shared between smoking initiation and schizophrenia and bipolar disorders [20]. It is noteworthy that the polygenic scores utilised in that study were computed from smaller summary statistics for schizophrenia (36,989 cases and 113,075 controls) and bipolar disorders (7481 cases and 9250 controls) compared to polygenic scores used in the present study. Indeed, our power calculation showed that the polygenic score for schizophrenia employed in the present analyses was substantially more powerful explaining 13.5% of the variance in schizophrenia (*p* = 3.89 × 10^− 213^); though, the polygenic score for bipolar disorders employed in the present study was comparable to the previous report [20]. Thus, it is feasible that the differences in the results, at least those related to polygenic predisposition to schizophrenia, may be due to powers accumulated in PGSs across studies. While there is a genetic correlation between smoking behaviours and schizophrenia [19], our results may imply that this correlation does not translate into the actual behavioural trait in older adults.

There is growing support for an association between smoking and depression; though, there is no consensus on the nature of this relationship. On one hand, it is feasible that smoking causes depression onset, on the other, it has been argued that depression causes smoking initiation [31] by relieving symptoms related to depression or reduction of the side effects of psychopharmacological treatments [32]. Our results showed that having started to smoke, older adults with a higher polygenic predisposition to major depressive disorders were more likely to become moderate to heavy smokers, which was used as a proxy for nicotine dependence [33]. This is in line with the findings from Virginia Twin Registry suggesting that genetic factors associated with an increased risk for depression overlap with those that convey a risk for daily smoking [34]. Our results further reiterate that familial vulnerability to depression, even in the absence of any history of depression, is likely to increase vulnerability to progressing to heavier smoking [35].

Similarly, to smoking initiation, we did not observe significant relationships of polygenic predisposition to schizophrenia, bipolar disorders, major depression, and intelligence with smoking cessation in older adults. The observed non-significant associations may reflect attrition effects, which are unavoidable in longitudinal cohorts. Similarly, because the results presented in the study are based on a longitudinal study with prospectively collected data, collider bias may have contributed to this finding [36], which might have arisen from selection bias or attrition. Even though comparisons with the national census showed that the ELSA sample was representative of the non-institutionalised general population aged ≥ 50 residing in the UK [37], to ensure we completely minimised any issues related to the selection bias we used inverse probability weighting in our models [38]. It is further feasible that single genetic markers for schizophrenia, bipolar disorders, major depression, and intelligence of large effects, rather than an aggerate of common genetic markers of small effect, influence the risk of smoking initiations in the general population. For example, one of the robust loci associated with schizophrenia identified by a large genome-wide association study meta-analysis [17] has been found in a gene cluster encoding neuronal nicotinic acetylcholine receptors (nAChR), which is linked to the heaviness of smoking in the general population [10, 39]. However, by design, polygenic scores do not capture other structural variants beyond common genetic markers of relatively small effects, such as rare variants, poorly tagged or multiple independent variants, gene-by-gene interactions and gene-environment correlations [40]. Nonetheless, our results have clear implications for public health; specifically because smoking initiation and cessation do not appear to be behaviours that share a common genetic ground with schizophrenia, bipolar disorders, major depression, and intelligence, it may be postulated that these results underscore the importance of smoking preventative campaigns and educational efforts directed towards the public about the harms of smoking.

### Limitations

Most smoking is initiated early in life, and the situation of participants may have been rather different then. Another limitation of the current study is that it uses cross-sectional data. Many of the respondents who were not smoking at the time of the survey will have relapsed afterwards. PGSs are fundamentally dependent on the availability of the summary statistics from genome-wide association studies, which are currently predominately based on European participants [41]. Therefore, it is essential to develop PGSs models in non-white populations before the results could be generalised to the whole population. Although we assessed several associations, which may raise some concerns over multiple statistical testing, our sample size was large enough to withstand multiple testing without increasing the risk of false positive results. It has been argued that adjusting for multiple statistical testing has significant disadvantages [42]. Consequently, rather than adjusting our *p*-values for multiple testing, we followed the new guidelines for statistical reporting [43] when presenting our results.

## Conclusion

We demonstrated that smoking is a behaviour that does not appear to share common genetic ground with schizophrenia, bipolar disorders, and intelligence in older adults. Once started to smoke, older adults with a higher polygenic predisposition to major depressive disorders are more likely to progress to heavier smoking, implying that these adults may require targeted smoking cessation services. The next steps in this line of research could be to investigate the response to antismoking interventions in those with a high polygenic predisposition to major psychiatric disorders.

## Methods

### Study Design and participants

We utilised data from the English Longitudinal Study of Ageing (ELSA), which is an ongoing large, multidisciplinary study of a nationally representative sample of the English population aged ≥ 50 years [37]. The ELSA study started in 2002 (wave 1) with participants recruited from an annual cross-sectional survey that was designed to monitor the health of the general population, who were then followed up every 2 years. The ELSA sample is periodically refreshed with younger participants to ensure that the full age spectrum is maintained [37]. Compared with the national census, the ELSA sample is representative of the non-institutionalised general population aged ≥ 50 residing in the UK [37]. Because the blood samples (for genetic data) were collected by nurses during a home visit at wave 2 (2004–2005) for the core members who started at wave 1, or wave 4 (2008–2009) for the participants joining the study at wave 4 through the refreshment sample, the data from these waves formed our analytic sample. Some of the ELSA participants reported a prior history of a mental illness; because we did not know if the onset of the mental illnesses preceded the initiation of smoking, we excluded participants who reported a previous history of psychiatric conditions including schizophrenia, bipolar disorders, and hallucinations (*n* = 798). This exclusion further allowed us to avoid detecting associations related to the presence of diagnoses of any of the major psychiatric conditions (rather than due to biological pleiotropy). Ethical approval for each of the ELSA waves was granted by the National Research Ethics Service (London Multicentre Research Ethics Committee). All participants gave informed consent.

### Outcomes

Smoking initiation and smoking cessation were defined based on responses to two questions: (1) “Have you ever smoked cigarettes?” (used to define smoking initiation) and (2) “Do you smoke cigarettes at all nowadays?” Participants who responded “yes” to the first question and “no” to the second one were classified as former smokers. The average number of cigarettes smoked per day was also recorded [44]. The heaviness of cigarettes smoking was assessed as the average number of cigarettes smoked a day among those participants who reported having ever smoked or being smokers at the time of the assessment, which was then categorised into two groups: (1) light smoker (< 10 cigarettes a day), and (2) moderate-heavy smokers (≥ 10 cigarettes a day). This measure has been validated against salivary cotinine levels in the Health Survey for England [45].

### Genetic data

*Quality control*. The genome-wide genotyping was performed at University College London Genomics in 2013–2014 using the Illumina HumanOmni2.5 BeadChips (HumanOmni2.5-4v1, HumanOmni2.5-8v1.3), which measures ~ 2.5 million markers that capture the genomic variation down to 2.5% minor allele frequency (MAF). Single-nucleotide polymorphism (SNPs) were excluded if they were non-autosomal, MAF was < 1%, if more than 2% of genotype data were missing and if the assumption of Hardy-Weinberg Equilibrium under P < 10^− 4^ was not fulfilled. Samples were removed based on call rate (< 0.99), heterozygosity, and relatedness and if the recorded sex phenotype was inconsistent with genetic sex (Additional file 1: Table S1). Relatedness in the sample was estimated using the identical by descent (IBD) probabilities [46] where one of each pair of individuals with an IBD value of > 0.2 were excluded at random. To identify ancestrally homogenous analytic samples the ELSA genomic samples use a combination of both self-reported ethnicity and analyses of genetic ancestry. Genetic ancestry was estimated via comparison of participants’ genotypes to global reference populations using principal component analyses (PCA) [47]. Because PCA allows examining population structure in a cohort by determining the average genome-wide genetic similarities of individual samples, derived principal components (PCs) can be used to group individuals with shared genetic ancestry, to identify outliers, and as covariates, to reduce false positives due to population stratification [47]. Although up to 98% of the ELSA participants self-described to be of European cultural background, PC highlighted the presence of ancestral admixture in n = 65 (0.9%) individuals (implying these individuals had ancestors from two or more populations [47]. Because accounting for systematic differences in allele frequencies is necessary for genetic analyses [47], these participants with ancestral admixture were removed from the analyses. The final sample includes all self-reported European participants that had PC loadings within ± one standard deviation of the mean for eigenvectors one.

To improve genome coverage, we imputed untyped quality-controlled genotypes to the Haplotype Reference Consortium [48, 49] using the University of Michigan Imputation Server [48] using IMPUTE2 for imputation. Post-imputation, we kept variants that were genotyped or imputed at INFO > 0.80, in low linkage disequilibrium (*R*^2^ < 0.1) and with Hardy-Weinberg Equilibrium *p*-value > 10^− 5^ giving us a total of 7,179,780 variants for further analyses. To investigate population structure, we use principal components analysis [47] implemented in PLINK 1.9 [50] retaining the top 10 principal components; these 10 principal components are depicted in Additional file 1: Figure S1. To ensure that the results obtained in the present study are not biased due to population stratification, all association analyses will be adjusted for principal components [47, 51].

*Polygenic score (PGS) analyses*. To calculate the polygenic score for schizophrenia (SZ-PGS), bipolar disorders (BD-PGS), major depressive disorders (MDD-PGS) and intelligence (IQ-PGS), we used the summary statistics from the latest genome-wide association studies for schizophrenia (*n*_cases_=69,369 and *n*_controls_=236,642), bipolar disorders (*n*_cases_=20,352 and *n*_controls_=31,358), major depressive disorders (*n*_cases_=135,458 and *n*_controls_=344,901) and intelligence (*n*_total_=269,867)[21–23, 52]. We calculated PGSs as a sum of the allele dosages, summing over the common markers which were within a certain p-value threshold (*p*_T_) (i.e., 0.001, 0.01, 0.05, 0.1, 0.3, 1), and weighted according to the strength of their effect estimate using PRSice [53]. To make a decision on which *p*_T_ for each PGS to take forward for further analyses, using the information on sample size (*n*), the total number of independent markers in genotyping panel (*m*) and lower and upper p-values (used to select markers into each PGS), we estimated the predictive accuracy (*R*^2^, *p*-value) for SZ-PGS, BD-PGS, MDD-PGS and IQ-PGS at each *p*_T_ (Additional file 1: Table S2) using Avengeme package implemented in R [54]. Our estimates showed that for SZ-PGS the ultimate *p*_T_ was 0.05 (*m* = 252,128, *R*^2^ = 0.135, *p* = 3.89 × 10^− 213^); here, the observed *R*^2^ = 0.135 is in line with multiple studies demonstrated that the polygenic score for schizophrenia explains between 13 and 25% of genetic liability to schizophrenia [17, 52]. For BD-PGS (*m* = 7220, *R*^2^ = 0.001, *p* = 0.038), MDD-PGS (*m* = 13,327, *R*^2^ = 0.007, *p* = 2.17 × 10^− 11^) and IQ-PGS (*m* = 27,912, *R*^2^ = 0.006, *p* = 3.56 × 10^− 10^) the best *p*_T_ was 0.001 (Additional file 1: Table S3). The SZ-PGS, BD-PGS, MDD-PGS and IQ-PGS at these p*-*value thresholds have a moderate degree of correlation (Additional file 1: Table S3). To aid the interpretability of the results, SZ-PGS, BD-PGS, MDD-PGS and IQ-PGS were centred by subtracting the mean and dividing by their corresponding standard deviations [20].

### Statistical analysis

To investigate the relationships of PGSs with each smoking-related behaviour, we employed binary logistic regression. All models were adjusted for age at the time of the assessment, sex, and genetic ancestry as measured with the top 10 principal components as covariates. Given the wide range of our participants’ ages, the impact of additional years from the baseline age may not have a stable linear increase for the log odds. Therefore, to capture the non-linear effects of ageing, we further included age^2^ and age^3^ as covariates. Interaction between sex and each PGS was tested by including a PGS × sex term in models. Interaction between PGS and age was similarly tested by including a PGS × age term in the models. However, these interactions were non-significant; therefore, the models were not stratified by sex or age; the final models were run without the interactions (i.e., PGS × sex and PGS × age). All analyses were weighted for non-response to requests for blood collections to wave 2 for the core members, or wave 4 for the participants who joined the study at wave 4 through the refreshment sample to ensure we minimised any issues related to the selection bias [38]. Odds ratios (ORs) are reported for one unit change in PGS. Association analyses were conducted in Stata release 14 (STATA Corp LP, USA).

## Electronic supplementary material

Below is the link to the electronic supplementary material.

**Supplementary Figure 1**. Depicts distribution of 10 principal components once 65 individuals with ancestral admixture were removed from the sample. **Supplementary Table 1**. An overview of the summary of full quality control procedure employed in the ELSA study and how many variants and/or participants were lost at each step. **Supplementary Table 2**. Estimated the predictive accuracy (R2, p-value) for SZ-PGS, BD-PGS, MDD-PGS and IQ-PGS at each pT. **Supplementary Table 3**. Correlations between each unstandardised polygenic score included in the analyse.

## Data Availability

The English Longitudinal Study of Ageing (ELSA) was developed by a team of researchers based at University College London, the Institute for Fiscal Studies and the National Centre for Social Research. The datasets generated and/or analysed during the current study are available in UK Data Services and can be accessed at: https://discover.ukdataservice.ac.uk. No administrative permissions were required to access these data.

## List of abbreviations

PGS: Polygenetic scores
GWAS: Genome-wide association studies
ELSA: English Longitudinal Study of Ageing
QC: Quality control
SNP: Single-nucleotide polymorphism
LD: Linkage disequilibrium
SD: Standard deviation
OR: Odds rations
SE: Standard errors
CI: Confidence interval
P: P-value
