# Supplementary material for: The shared genetic architecture of smoking behaviours and psychiatric disorders: evidence from a population-based longitudinal study in England
Source: BMC Genom Data. 2023 May 30;24:31. doi: 10.1186/s12863-023-01131-8 (PMC10230674; doi:10.1186/s12863-023-01131-8)
Supplement: Supplementary file 1 — Supplementary Figure 1. Depicts distribution of 10 principal components once 65 individuals with ancestral admixture were removed from the sample. Supplementary Table 1. An overview of the summary of full quality control procedure employed in the ELSA study and how many variants and/or participants were lost at each step. Supplementary Table 2. Estimated the predictive accuracy (R2, p-value) for SZ-PGS, BD-PGS, MDD-PGS and IQ-PGS at each pT. Supplementary Table 3. Correlations between each unstandardised polygenic score included in the analyse. [file 12863_2023_1131_MOESM1_ESM.docx]

**Supplementary Figure 1. Depicts distribution of 10 principal components once 65 individuals with ancestral admixture were removed from the sample.**


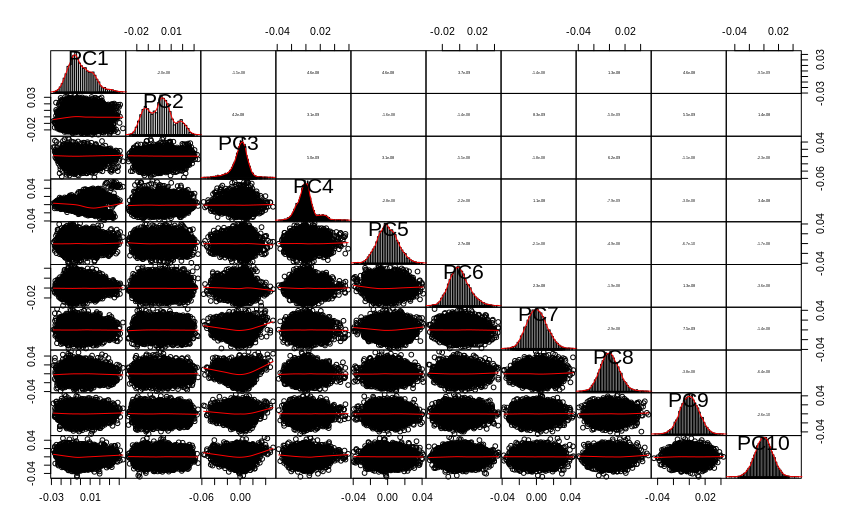


**Supplementary Table 1.** An overview of the summary of full quality control procedure employed in the ELSA study and how many variants and/or participants were lost at each step

| Quality Control steps in ELSA | | | |  |
| --- | --- | --- | --- | --- |
| *Lost due to SNP-based QC* | | *n (SNPs)* | % | |
|  | Missing SNPs (0.02) | 41614 | 1.9 | |
|  | Autosomal SNPs | 48578 | 2.2 | |
|  | MAF 0.01 | 759972 | 34.1 | |
|  | Update rsids | 2284 | 0.1 | |
|  | HWE (0.0001) | 6079 | 0.3 | |
|  |  |  |  | |
|  | *Total removed* | *858527* | *38.5* | |
|  | *Total remaining* | *1372240* | *61.5* | |
|  |  |  |  | |
| *Lost due to Individual-based QC* | | *n (individuals)* | % | |
|  | Missingness (0.02) | 39 | 0.5 | |
|  | Heterogeneity | 76 | 1.0 | |
|  | Sex discordance | 5 | 0.1 | |
|  | Ancestry outliers | 64 | 0.9 | |
|  | Relatedness/Duplicates | 5 | 0.1 | |
|  | Unique IDs are not present | 41 | 0.5 | |
|  |  |  |  | |
|  | *Total removed* | *229* | *3.1* | |
|  | *Total remaining* | *7183* | *96.9* | |

HWE, Hardy-Weinberg equilibrium; MAF, minor allele frequency; SNP, single nucleotide polymorphisms

**Supplementary Table 2.** **Estimated the predictive accuracy (*R*^2^, *p*-value) for SZ-PGS, BD-PGS, MDD-PGS and IQ-PGS at each *p*_T_**

|  | | ***P*-value threshold for Polygenic scores** | | | | | |
| --- | --- | --- | --- | --- | --- | --- | --- |
|  |  | **0.001** | **0.01** | **0.05** | **0.1** | **0.3** | **1** |
|  |  |  |  |  |  |  |  |
| **SZ-PGS** | m | 42964 | 109480 | **252128** | 379025 | 771994 | 1862381 |
|  | *R*^2^ | 0.030 | 0.020 | **0.135** | 0.015 | 0.013 | 0.07 |
|  | *p* | 2.23×10^-44^ | 6.53×10^-29^ | **3.89×10^-213^** | 3.26×10^-23^ | 1.50×10^-19^ | 2.50×10^-11^ |
|  |  |  |  |  |  |  |  |
| **BD-PGS** | m | **7220** | 31749 | 103500 | 177678 | 435525 | 1228480 |
|  | *R*^2^ | **0.001** | 0.0003 | 0.0003 | 0.0003 | 0.0002 | 8.84×10^-05^ |
|  | *p* | **0.038** | 0.079 | 0.092 | 0.102 | 0.137 | 0.213 |
|  |  |  |  |  |  |  |  |
| **MDD-PGS** | m | **13327** | 44993 | 125058 | 201806 | 454098 | 1197733 |
|  | *R*^2^ | **0.007** | 0.003 | 0.003 | 0.003 | 0.002 | 0.001 |
|  | *p* | **2.17×10^-11^** | 1.58×10^-06^ | 1.21×10^-05^ | 2.45×10^-05^ | <0.0001 | 0.007 |
|  |  |  |  |  |  |  |  |
| **IQ-PGS** | m | **27912** | 73878 | 170072 | 257535 | 526713 | 1269550 |
|  | *R*^2^ | **0.006** | 0.005 | 0.006 | 0.006 | 0.005 | 0.003 |
|  | *p* | **3.56×10^-10^** | 4.55×10^-09^ | 1.37×10^-09^ | 1.62×10^-09^ | 2.40×10^-08^ | 3.62×10^-05^ |

*M,* total number of independent markers in genotyping panel; P, p-value

SZ-PGS, polygenic score for schizophrenia; BD-PGS, polygenic score for bipolar disorder; MDD-PGS, polygenic score for major depressive disorder; IQ-PGS

**Supplementary Table 3**. Correlations between each unstandardised polygenic score included in the analyse

|  | SZ-PGS | BD-PGS | MDD-PGS | IQ-PGS |
| --- | --- | --- | --- | --- |
| SZ-PGS | 1.00 |  |  |  |
| BD-PGS | 0.38, P<0.011 | 1.00 |  |  |
| MDD-PGS | 0.67, P<0.001 | 0.32, P<0.001 | 1.00 |  |
| IQ-PGS | -0.55, P<0.001 | -0.20, P<0.001 | -0.45, P<0.001 | 1.00 |

SZ-PGS, polygenic score for schizophrenia; BD-PGS, bipolar disorders; MDD-PGS, major depressive disorder; IQ-PGS, intelligence.
